# Immature Teratoma after Three Laparoscopic Resections for Mature Cystic Teratomas

**DOI:** 10.1155/2014/264959

**Published:** 2014-05-12

**Authors:** Kazuhiro Nishioka, Naoto Furukawa, Taketoshi Noguchi, Hirotaka Kajihara, Kiyoshige Horie

**Affiliations:** ^1^Department of Obstetrics and Gynecology, Yamato Takada Municipal Hospital, 1-1 Isonokita-cho, Yamato Takada, Nara 635-8501, Japan; ^2^Department of Obstetrics and Gynecology, Nara Medical University, 840 Shijo-cho, Kashihara, Nara 634-8522, Japan

## Abstract

We report a case in which an immature teratoma developed following three previous resections for mature cystic teratomas. The patient was a 26-year-old nulliparous woman with a regular menstrual cycle. Twelve years earlier, she had consulted a pediatrician for complaints of lower abdominal pain. Bilateral cystic teratomas were suspected and she underwent a left salpingo-oophorectomy and a right cystectomy laparoscopically, and bilateral mature cystic teratomas were diagnosed histologically. She underwent a right cystectomy twice afterwards and mature cystic teratomas were diagnosed. Three years after the third surgery, a regular checkup performed annually for ovarian cyst recurrence revealed a 9.3 cm ovarian cyst by ultrasonography without marker elevation or complaint of symptoms. Magnetic resonance imaging (MRI) showed a 10 cm multilocular cyst, including a part with heterogeneous medium and high-signal intensity on T2-weighted images, which revealed enhancement on dynamic contrast-enhanced MRI unlike the previous images. Ovarian tumors, including immature teratomas and malignancy, were considered. She had a strong wish to undergo laparoscopic surgery. She was diagnosed with an immature teratoma, grade 1 of the right ovary. Although the frequency of recurrence of immature teratomas after resection of mature cystic teratomas is very low, regular checkups are necessary because there may be no associated symptoms.

## 1. Introduction

Teratomas are the most common type of ovarian germ cell neoplasm, comprising approximately 15% of all ovarian tumors. They occur within a very wide age range but usually occur during the reproductive years. Mature cystic teratomas are rarely large (typically <10 cm) and often bilateral (15%–25%) [1]. Microscopically, they are usually unilocular cysts containing tissue from all three germ cells and sometimes containing teeth, bone, and neural tissue [2]. Management of teratomas depends on the symptoms and tumor size. Conservative management and surgery, including laparoscopic cystectomy and oophorectomy, are usually selected. The frequency of benign mature cystic teratomas arising after resection for mature cystic teratomas is 3%, and the frequency of malignant degeneration is 2% [3]. When malignant degeneration occurs, the most common secondary tumor is a squamous cell carcinoma, which is usually found in women aged more than 50 years [4], but these are rarely immature teratomas. Here we report a case in which an immature teratoma developed following three previous resections for mature cystic teratomas.

## 2. Case Presentation

The patient was a 26-year-old nulliparous woman with a regular menstrual cycle. Twelve years earlier she had consulted a pediatrician for complaints of lower abdominal pain. Ultrasonography revealed a giant pelvic cyst, mostly echogenic, which was larger than fist size. Carbohydrate antigen 19-9 (CA19-9) and squamous cell carcinoma antigen (SCC) were elevated to 169 U/mL and 16 ng/mL, respectively. Magnetic resonance imaging (MRI) showed a large 12 cm unilocular tumor on the left ovary and a 4 cm tumor on the right ovary with high-signal intensity on T1-weighted images and signal dropout on fat suppression imaging. Bilateral cystic teratomas were suspected and she underwent a left salpingo-oophorectomy and a right cystectomy laparoscopically, and bilateral mature cystic teratomas were diagnosed histologically.

**Figure 1:**
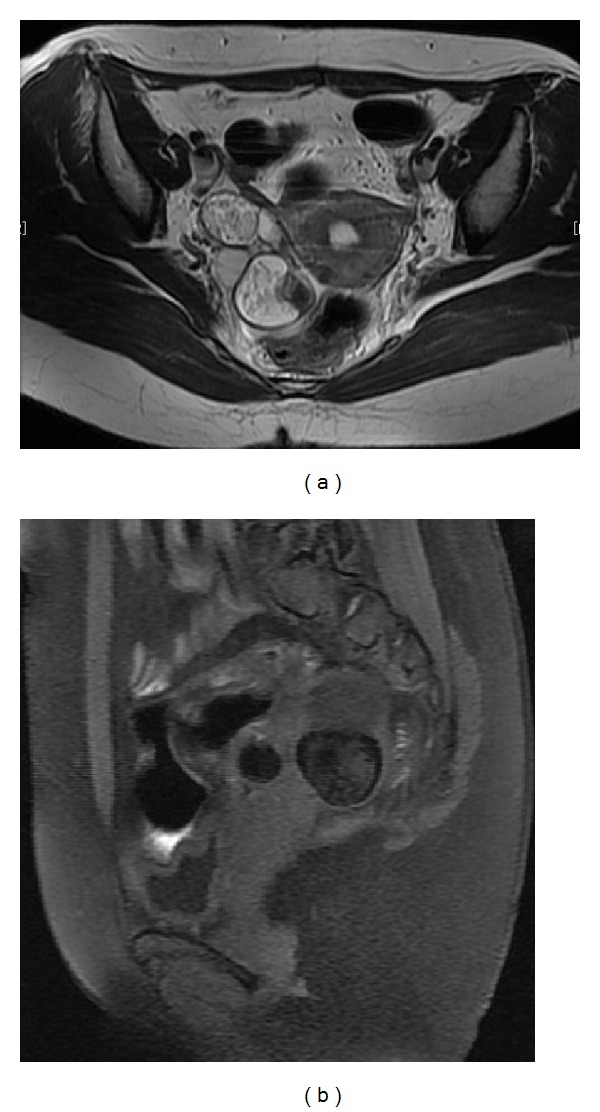
Right ovarian cyst with fat in cystic cavity before second surgery. (a) Axial T2-weighted image shows high-signal intensity cyst. (b) Fat-saturated T1-weighted image shows signal decrease on the cyst.

**Figure 2:**
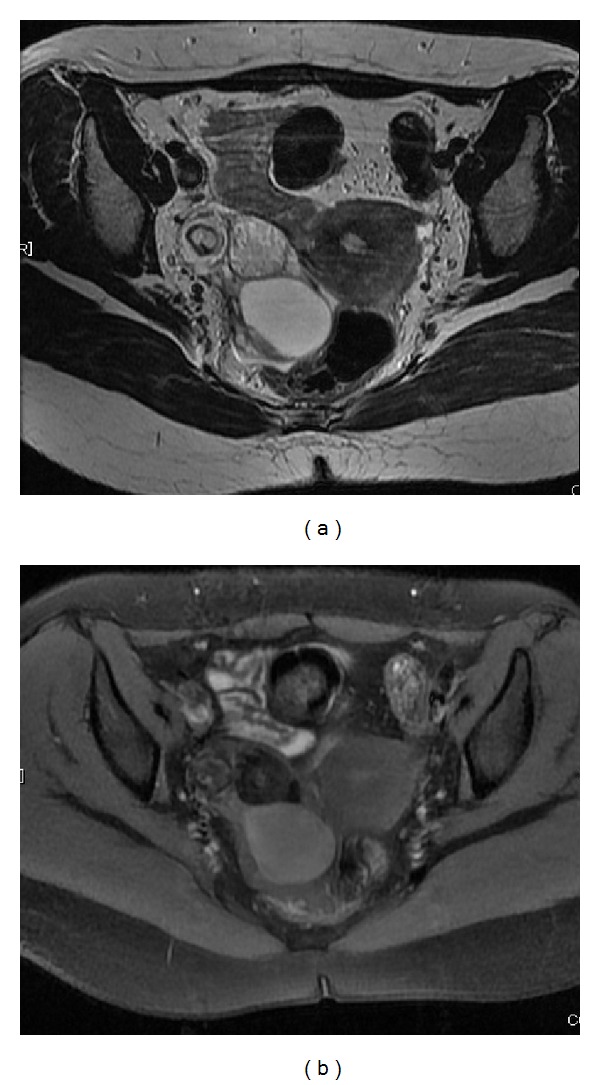
Right ovarian cyst with fat in the cystic cavity before the third surgery. The cyst size was the same as the cyst size before the second surgery. (a) Axial T2-weighted image shows high-signal intensity cyst. (b) Fat-saturated T1-weighted image shows a signal drop on the cyst.

**Figure 3:**
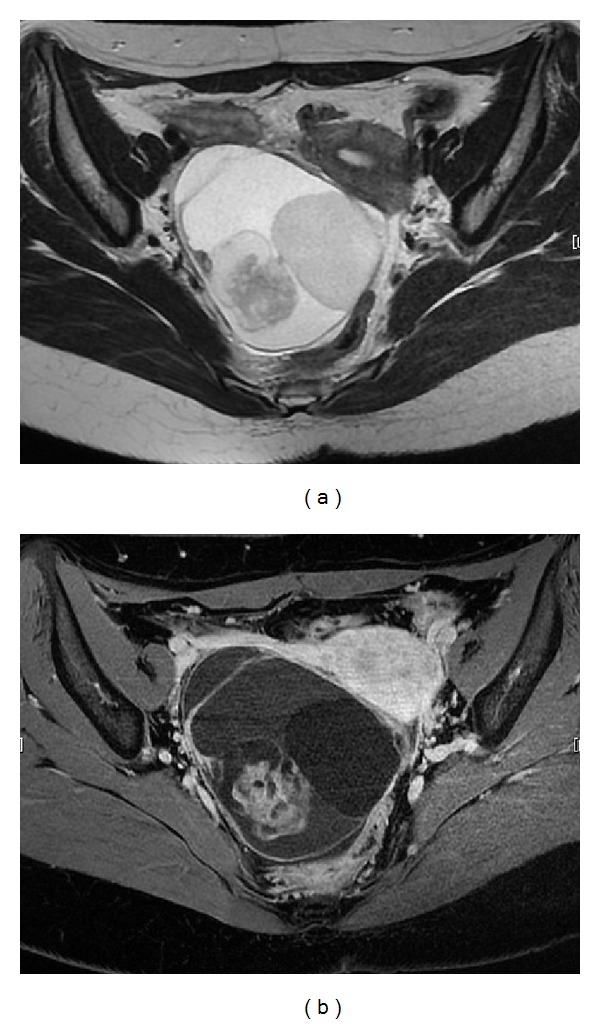
Right ovarian tumor with a solid part in the cystic cavity before the fourth surgery. The tumor size was greater than that before the third surgery. (a) Axial T2-weighted image shows high-signal intensity mass. (b) Gadolinium-enhanced T1-weighted image shows focal enhancement of the tumor.

**Figure 4:**
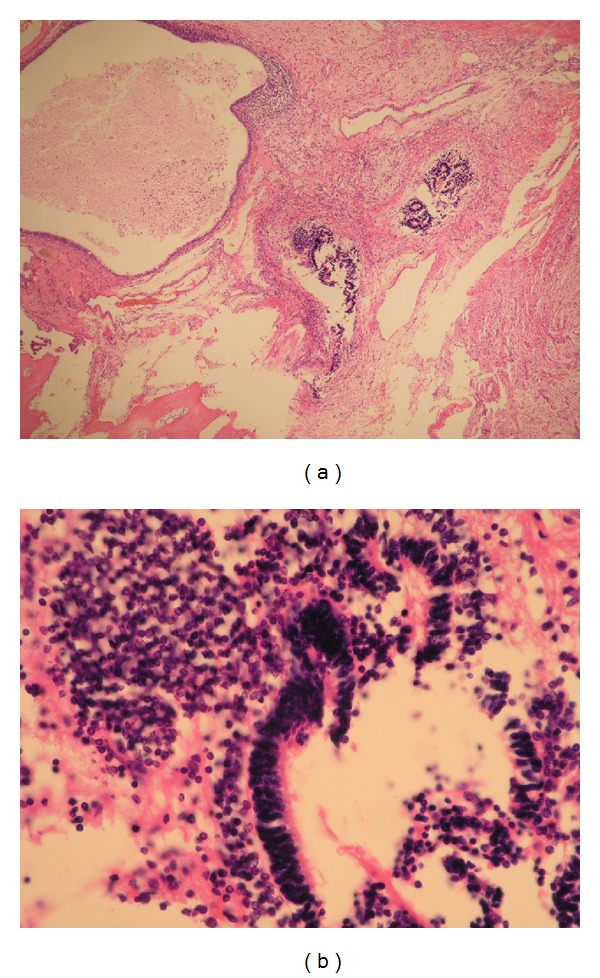
Pathologic findings of right ovary. Immature neuroepithelium seen with hair follicles and sebaceous glands. (a) H&E ×40. (b) H&E ×400.

CA19-9 and SCC values were in the normal range after tumor resection. Eight years after the first surgery, a regular annual checkup for ovarian cyst recurrence revealed swelling of the right ovary by ultrasonography, without elevated CA19-9 and SCC. The patient had no complaints. MRI showed a 6.5 cm multilocular ovarian cyst with signal decrease on fat-saturated T1-weighted images and these findings were consistent with recurrence of cystic teratoma (Figure 1). She underwent right cystectomy laparoscopically and was diagnosed with mature cystic teratoma of the right ovary. At 17 months after the second surgery, swelling of the right ovary was detected and recurrence was suspected. MRI showed a multilocular 7.0 cm ovarian cyst (Figure 2). She underwent laparoscopic cystectomy of the right ovary and histologic examination revealed a mature cystic teratoma. Three years after the third surgery, a regular annual checkup for ovarian cyst recurrence revealed a 9.3 cm ovarian cyst by ultrasonography without marker elevation or complaint. MRI showed a 10 cm multilocular cyst, including a part with heterogeneous medium and high-signal intensity on T2-weighted images, which revealed enhancement on dynamic contrast-enhanced MRI, unlike the previous images (Figure 3). Based on the MRI, ovarian tumor, including immature teratoma and malignancy, was considered, and she had a strong wish to undergo laparoscopic surgery. Therefore, a laparoscopy was performed, revealing right ovary swelling, 10 cm in size, without ascites or other abnormal intra-abdominal findings. The tumor was excised, placed into a bag, reduced by sucking fluid from the tumor, and removed through the umbilical incision. Pathologic examination revealed various mature tissues with focal immature neuroepithelial cells forming rosette-like structures (Figure 4), leading to the diagnosis of an immature teratoma, grade 1 according to Thurlbeck and Scully's histogrognostic scoring, as modified by Norris et al. [5]. Her menstrual cycle was regular after the last surgery.

## 3. Discussion

Immature teratomas are uncommon (1% of ovarian teratomas) and affect younger women [6, 7]. There is no definite diagnostic criterion for immature teratomas in MRI. Some researchers report that a large, irregular solid component containing coarse calcifications and small foci of fat is common and intralesional fat is typically observed as punctate foci scattered throughout the tumor in contrast to the more uniform appearance of liquid sebum in a mature cystic teratoma [8, 9]. In the present case, these findings were not observed. The mean tumor size of the immature teratomas was 15 cm, which is significantly larger than the typical reported size of mature teratomas, 7.5 cm [10]. Moreover, immature teratomas may be solid or have a prominent solid component with a cystic element. In the present case, the tumor grew larger at every recurrence, and a solid part emerged in the last recurrence. Therefore, an immature teratoma was initially suspected. No symptoms, however, were detected. Symptoms of ovarian tumors relate to tumor size. The incidence of symptoms in tumors with a mean size of 7.5 cm was significantly different from that of tumors with a mean size of 15.3 cm (24% versus 92%) [10]. Mature cystic teratomas are associated with the future development of ovarian germ cell tumors in patients treated with resection of mature cystic teratomas [3, 11]. The incidence of the recurrence of germ cell tumors is 3.4% to 5.1%, and bilateral or multiple cysts might be a predictive factor of recurrence. Recurrence of immature teratomas is very rare, 1% [3]. In the present case, although bilateral and unilocular cysts were initially observed, a multilocular cyst was observed upon recurrence. The mean time interval until the development of recurrence of mature cystic teratomas is 9 years and for malignant germ cell tumors it is 2 years [3]. In the present case, there was a 3-year interval after the third surgery, suggesting that the interval until the development of recurrence in low malignant potential tumors, such as immature teratoma G1, is also earlier than in mature cystic teratomas. In the present case, the tumor was resected laparoscopically. Surgical rupture of the tumor may cause gliomatosis peritonei. Gliomatosis peritonei is relatively rare and not reported to have a poor prognosis when associated with an immature teratoma [12].

## 4. Conclusion

This brief report presents the case of a patient with an immature teratoma that developed after three previous surgeries for mature cystic teratomas. Although the frequency of recurrence is very low, regular checkups are necessary because there may be no associated symptoms.
